# Control of pyrethroid and DDT-resistant *Anopheles gambiae *by application of indoor residual spraying or mosquito nets treated with a long-lasting organophosphate insecticide, chlorpyrifos-methyl

**DOI:** 10.1186/1475-2875-9-44

**Published:** 2010-02-08

**Authors:** Raphael N'Guessan, Pelagie Boko, Abibathou Odjo, Joseph Chabi, Martin Akogbeto, Mark Rowland

**Affiliations:** 1 Department of Infectious & Tropical Diseases, London School of Hygiene and Tropical Medicine, Keppel Street, London, WC1E 7HT, UK; 2 CREC laboratories, Centre de Recherche Entomologique de Cotonou, Laboratoire Nationale, Ministère de la Santé, Cotonou 06 BP 2604, Benin

## Abstract

**Background:**

Scaling up of long-lasting insecticidal nets (LLINs) and indoor residual spraying (IRS) with support from the Global Fund and President's Malaria Initiative is providing increased opportunities for malaria control in Africa. The most cost-effective and longest-lasting residual insecticide DDT is also the most environmentally persistent. Alternative residual insecticides exist, but are too short-lived or too expensive to sustain. Dow Agrosciences have developed a microencapsulated formulation (CS) of the organophosphate chlorpyrifos methyl as a cost-effective, long-lasting alternative to DDT.

**Methods:**

Chlorpyrifos methyl CS was tested as an IRS or ITN treatment in experimental huts in an area of Benin where *Anopheles gambiae *and *Culex quinquefasiactus *are resistant to pyrethroids, but susceptible to organophosphates. Efficacy and residual activity was compared to that of DDT and the pyrethroid lambdacyalothrin.

**Results:**

IRS with chlorpyrifos methyl killed 95% of *An. gambiae *that entered the hut as compared to 31% with lambdacyhalothrin and 50% with DDT. Control of *Cx. quinquefasciatus *showed a similar trend; although the level of mortality with chlorpyrifos methyl was lower (66%) it was still much higher than for DDT (14%) or pyrethroid (15%) treatments. Nets impregnated with lambdacyhalothrin were compromized by resistance, killing only 30% of *An. gambiae *and 8% of *Cx. quinquefasciatus*. Nets impregnated with chlorpyrifos methyl killed more (45% of *An gambiae *and 15% of *Cx. quinquefasciatus*), but its activity on netting was of short duration. Contact bioassays on the sprayed cement-sand walls over the nine months of monitoring showed no loss of activity of chlorpyrifos methyl, whereas lambdacyhalothrin and DDT lost activity within a few months of spraying.

**Conclusion:**

As an IRS treatment against pyrethroid resistant mosquitoes chlorpyrifos methyl CS outperformed DDT and lambdacyhalothrin. In IRS campaigns, chlorpyrifos methyl CS should show higher, more-sustained levels of malaria transmission control than conventional formulations of DDT or pyrethroids. The remarkable residual activity indicates that cost-effective alternatives to DDT are feasible through modern formulation technology.

## Background

International efforts to control malaria supported by the Global Fund and the President's Malaria Initiative (PMI) are underway in many parts of Africa using strategies based on the scaling-up of long-lasting insecticidal nets (LLINs) and indoor residual spraying (IRS) [1-3]. Pyrethroids are the only group of insecticides currently recommended for use on mosquito nets [4]. Pyrethroid resistance has, in recent years, become widespread among anopheline mosquitoes in western and southern Africa and also occurs in eastern and central Africa [5-8]. The recent evolution and spread of pyrethroid resistance in West Africa in the Mopti (M) molecular form of *Anopheles gambiae *sensu stricto presents a grave threat to control because carriers of this particular resistance are not killed by pyrethroid-treated nets or residual spraying [9,10]. With the scaling-up of malaria control efforts the continued selection of pyrethroid resistance [11,12] may compromise malaria control programmes and render this group of insecticides ineffective. It is important to investigate alternative insecticides on nets to supplement existing pyrethroid-based LLINs.

The target site insensitivity gene that confers knock down resistance *kdr *to pyrethroids in *An. gambiae *shows cross resistance to DDT [6]. Of the insecticides recommended by WHO for IRS, the most long-lasting and cost-effective is DDT [13]. No assessment of DDT has been made in areas where *kdr *is prevalent. A small-scale trial of DDT is essential before any decision to redeploy DDT can be made in West Africa.

Because of DDT's damaging environmental impact, the Stockholm Convention on Persistent Organic Pollutants stipulates that 'countries are encouraged to reduce and eliminate the use of DDT over time and switch to alternative insecticides' [14]. The alternative classes of insecticide to DDT - the organophosphates, carbamates and pyrethroids - are more expensive and shorter-lived. For programmes that use DDT to remain viable it is necessary to develop long-lasting formulations of the alternative insecticides before DDT can be replaced [15]. Dow Agrosciences has among its portfolio of insecticides the organophosphate chlorpyrifos methyl which is effective against anophelines but applied as a wettable powder, the standard IRS formulation, it is too short-lived [16]. The company has, therefore, developed a microencapsulated formulation to improve residual activity. Its limited environmental persistence [16] and lack of cross resistance makes chlorpyrifos methyl a more attractive prospect than DDT for IRS should re-formulation increase its residual life. It also has the advantage of not selecting for pyrethroid resistance or undermining LLIN use.

This paper reports on an experimental hut trial in southern Benin of microencapsulated chlorpyrifos methyl. Efficacy is compared with that of DDT and the pyrethroid lambdacyalothrin in an area where *An. gambiae *has become difficult to control with pyrethroids [9].

## Methods

### Study sites and experimental huts

The evaluation was carried out in experimental huts situated in Ladji, a peri-urban village on the periphery of Cotonou, the capital of Benin. The village floods during the rainy season, creating breeding sites for *An. gambiae*. The local *An. gambiae *population is comprised entirely of the M taxon and is resistant to pyrethroids and DDT, with *kdr *at high frequency and metabolic resistance also present [9,17]. The nuisance mosquito *Culex quinquefasciatus *is present year round and is resistant to pyrethroid, carbamate and organophosphate insecticides [17]. The experimental huts are made from concrete bricks, with roofs of corrugated iron, ceilings lined with plastic sheeting on the interior surface and walls plastered with a cement/sand mix [18-20]. Each hut stands on a concrete base surrounded by a water-filled moat to exclude scavenging ants. Entry of mosquitoes occurs via four slits, 1 cm wide, located on three sides of the hut. Mosquitoes exit into a verandah trap projecting from the fourth side.

### Insecticide treatments

The insecticides used were:

• chlorpyrifos methyl 24% CS ('Reldan GF 1246', Dow AgroSciences)

• DDT 50% WP (sourced by Dow from South Africa)

• lambdacyhalothrin 2.5% CS ('Icon', Syngenta, Switzerland) microencapsulation designed for ITNs.

• Lambdacyhalothrin 10% WP ('Icon' Syngenta, Switzerland) wettable powder designed for IRS.

The following treatments and application rates were compared in seven experimental huts:

• chlorpyrifos methyl IRS at 500 mg/m^2^

• DDT IRS at 2 g/m^2^

• Lambdacyhalothrin 10% WP, IRS at 30 mg/m^2^

• chlorpyrifos methyl ITN at 100 mg/m^2^

• lambdacyhalothrin 2.5% CS, ITN at 18 mg/m^2^

• unsprayed control hut

• untreated net

The Chlorpyrifos methyl dosage applied for IRS was recommended by Dow Agrosciences whereas the application rate for the ITN was the one used in experimental huts in Ivory Coast to control the Savanah (S) taxon of pyrethroid-resistant *An. gambiae *[21].

The dosages for DDT and lambdacyalothrin treatments are those conventionally used and recommended by WHO.

The test nets were made of 100-denier polyester netting in which a total of 80 holes of 4 cm^2 ^area were cut along each side and end panel to simulate wear and tear. Nets were treated with insecticide by hand dipping. Insecticide was sprayed onto interior walls and plastic sheeting using a Hudson compression sprayer equipped with a flat fan nozzle. The evaluation started one week after treatment and ran for 42 nights from 8 April to 24 June 2005.

### Study procedure

The two net treatments and the untreated control net were rotated between three of the huts at six-day intervals. The four huts dedicated for IRS treatment were fixed throughout the study and the treatments could not, of course, be rotated. The volunteer sleepers gave informed consent and were provided with chemoprophylaxis. They slept in the huts from 20:00 to 05:00 each night, and were rotated between huts on successive nights to adjust for any variation in attractiveness to mosquitoes. Mosquitoes were collected each morning at 5:00 from floors, walls, ceilings and verandahs, and transported to the laboratory for identification to species, mortality counts and determination of gonotrophic condition. Live mosquitoes were held in plastic cups and delayed mortality was recorded after 24 h. The effects of each treatment were expressed relative to the control in terms of:

• Deterrence: percentage reduction in the number of mosquitoes caught in treated hut relative to the number caught in the control hut;

• Induced exiting: percentage of the mosquitoes collected from the verandah trap of treated hut relative to percentage caught in verandah trap of control hut;

• Inhibition of blood-feeding: percentage of the mosquitoes collected which was blood fed in the treated hut relative to percentage blood-fed in the control hut;

• Induced mortality: percentage of dead mosquitoes in treated hut relative to percentage dead in control hut.

• If a treatment deters a significant number of mosquitoes from entering the hut, the values given by proportion blood feeding in the treatment hut may underestimate the full personal protective effect of the treatments. The personal protective effect of a treatment is better described by the reduction in the actual number of blood-fed mosquitoes in the treatment hut relative to the number blood-fed in the control hut:

where B_u _= is the total number of blood-fed mosquitoes in the untreated control huts and B_t _is the total number blood-fed in the huts with insecticide treatment.

### Residual activity of insecticide treatments

To assess residual activity on treated walls or nets cone bioassay tests were undertaken each month using 3-5 day old *An. gambiae *females of a laboratory susceptible strain (Kisumu). Mosquitoes were exposed to nets for 3 min or to sprayed walls for 30 min as per WHO guidelines [22]. Approximately 50 mosquitoes were used per test.

### Data analysis

Data were entered in Excel and transferred to STATA 6.0 software for further analysis. The numbers of mosquitoes collected each night and the actual numbers bloodfed was analysed per treatment using the Kruskal-Wallis and Wilcoxon rank sum tests. Proportional data (exiting rate, blood-feeding, mortality) were analysed using logistic regression after adjusting for the effect of sleeper and hut. A Chi-square test for trend was performed to assess whether there was any change in mortality over time in bioassay tests.

## Results

### Trials of ITNs against *An. gambiae *and *Cx. quinquefasciatus*

**Figure 1:**
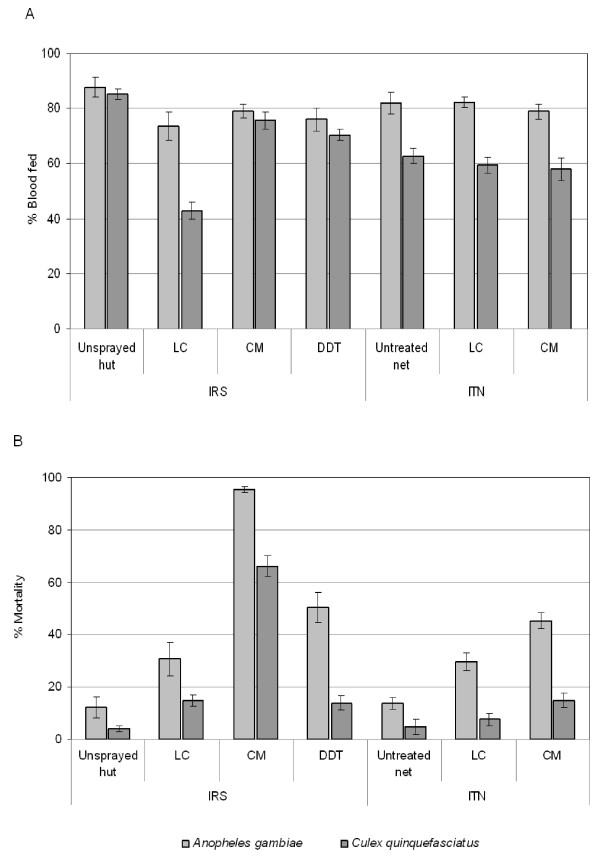
**Percentage of *Anopheles gambiae *and *Culex quinquefasciatus *bloodfed and dead in experimental huts**. Bloodfeeding (A) and mortality (B) rates with 95% confidence interval, of *An. gambiae *and *Cx. quinquefasciatus *in huts with different indoor residual spray (IRS) treatments and insecticide treated net (ITN). For each mosquito species within each intervention arm (IRS and ITN), treatments sharing the same letters in the middle of bars are not significantly different. LC = Lambdacyhalothrin; CM = Chlorpyrifos methyl; DDT = Dichlorodiphenyltrichloroethane

Table 1 shows the total number of *An. gambiae *and *Cx. quinquefasciatus *collected from the rooms with untreated or insecticide treated nets and the proportions exiting into the verandas. Figure 1A shows the percentage blood-fed and dying among the total collected. An average of 14 *An. gambiae *and 19 *Cx. quinquefasciatus *females were collected each morning from the rooms and verandas of the huts with nets. There was no significant difference in the number of *An. gambiae *collected between the untreated control and chlorpyrifos methyl huts (p = 0.49). By contrast, the number of *An. gambiae *found in the lambdacyhalothrin huts was 43.6% lower than in the untreated control huts (P < 0.001). Hence, there was no evidence of deterrence with the organophosphate chlorpyrifos methyl in contrast to the pyrethroid lambdacyhalothrin ITN. The trends with *Cx. quinquefasciatus *were similar to *An. gambiae*.

**Table 1:** Summary results of mosquitoes hut frequenting habit and exit rates in huts.

	Total entered	Average per night	Actual number fed	(%) personnel protection	% exiting (CI)
**ITNs**					
*Anopheles gambiae *s.s.					
Untreated net	689^a^	17.2	572	-	25.0^a ^(21.7-28.2)
LC	386^b^	9.7	317	44.6	29.0^a ^(24.5-33.5)
CM	648^a^	16.2	518	9.4	34.2^a ^(31.5-39.2)
					
*Culex quinquefasciatus*					
Untreated net	845^a^	21.1	531	-	29.8^a ^(26.7-32.9)
LC	598^b^	14.9	355	33.1	35.9^a ^(32.1-39.8)
CM	839^a^	21.0	487	8.3	34.0^a ^(30.6-37.1)
					
**IRS**					
*Anopheles. gambiae *s.s.					
Unsprayed hut	203^a^	5.1	178	-	45.8^a ^(39.8-52.7)
LC	117^a^	2.9	86	51.7	58.1^b ^(49.2-67.1)
CM	420^b^	10.5	336	0	50.5^a ^(43.4-58.5)
DDT	268^a^	6.7	201	0	41.3^a ^(36.0-47.7)
					
*Culex quinquefasciatus*					
Unsprayed hut	858^a^	21.4	730	-	52.7^a ^(49.3-56.0)
LC	769^a^	19.2	330	54.8	54.6^a ^(51.1-58.1)
CM	817^a^	20.4	621	14.9	51.0^a ^(47.6-55.2)
DDT	764^a^	19.1	535	26.7	49.7^a ^(44.1-55.0)

Summary results of the impact of insecticide treated nets (ITNs) and indoor residual spray (IRS) treatments on hut frequenting habit, individual exposure to bites and exit rates of *An. gambiae *and *Cx. quinquefasciatus *to the verandahs of the huts in Ladji. For each intervention arm (ITN and IRS) and mosquito species, values in columns sharing the same letter superscript do not differ significantly. LC = Lambdacyhalothrin; CM = Chlorpyrifos methyl; DDT = Dichlorodiphenyltrichloroethane

The chlorpyrifos-methyl- and lambdacyhalothrin-treated nets induced little or no additional exiting of *An. gambiae *or *Cx. quinquefasciatus *into the verandas over that recorded for untreated nets.

The proportions of *An. gambiae *and *Cx. quinquefasciatus *blood-feeding through the sides or holes of the chlorpyrifos-methyl ITN were not significantly different from the untreated control nets or lambdacyhalothrin treated nets (P > 0.05 for both species). However, the reduced entry rate of *An. gambiae *to hut with lambdacyhalothrin-treated net indicated a 44.6% lower risk of exposure to potential infective bites than in huts with control or chlorpyrifos methyl-treated nets (table 1). Hence, there was no evidence of blood-feeding inhibition with chlorpyrifos-methyl- or lambdacyhalothrin-treated nets though the pyretroid on net still procures some appreciable degree of personal protection against pyrethroid-resistant *An. gambiae *or *Cx. quinquefasciatus *in this area of Benin.

The percentage mortality among *An. gambiae *was 45.2% with the chlorpyrifos methyl-treated net and only 29.8% with the lambacyalothrin-treated net (Figure 1B). Mortality rates among *Cx. quinquefasciatus *were lower than among *An. gambiae *and did not exceed 15% with either type of treated net (Figure 1B).

### Trials of IRS against *An. gambiae *and *Cx. quinquefasciatus*

Table 1 shows the total number collected from the IRS treated rooms and the proportions exiting into the verandas. Figure 1B shows the percentage blood-fed and dying among the *An. gambiae *and *Cx. quinquefasciatus *collections. Owing to differences in site attractiveness of individual huts, which were located at different places within the village, and to the inability to rotate IRS treatments, it was not possible to interpret the overall numbers collected from the rooms in terms of treatment effects. Differences in hut position were a confounding source of error. By serendipity a significantly larger number of *An. gambiae *were collected from the chlorpyrifos-methyl treated hut than from the other types of hut (Table 1).

The percentages of *An. gambiae *and *Cx. quinquefasciatus *collected from the verandas of DDT, lambdacyhalothrin and chlorpyrifos methyl IRS treated huts were similar to those from the control verandas; the only exception was the significantly higher proportion of *An. gambiae*, which exited the lambdacyhalothrin treated hut (Table 1).

Blood-feeding rates of *An. gambiae *and *Cx. quinquefasciatus *in the chlorpyrifos methyl and DDT treated huts were not significantly different from the untreated control (Figure 1A). In the lambdacyhalothrin treated hut half as many *Cx. quinquefasciatus *were blood-fed compared to the control hut (P < 0.001). However a similar trend was not observed with *An. gambiae *in the lambdacyhalothrin treated hut.

Mortality of pyrethroid resistant *An. gambiae *was 95.5% with chlorpyrifos methyl IRS compared to 50.4% in the hut sprayed with DDT and 30.8% in the hut sprayed with lambdacyhalothrin (Figure 1B). The mortality of *Cx. quinquefasciatus *in the chlorpyrifos methyl IRS hut was 66.1% whereas in the DDT and lambdacyhalothrin IRS huts was only 14% (Figure 1B). Chlorpyrifos methyl IRS showed greater potential than DDT or lambdacyhalothrin IRS for control of pyrethroid resistant *An. gambiae *M form and *Cx. quinquefasciatus *in areas of high *kdr *frequency.

Neither chlorpyrifos methyl nor lambdacyhalothrin stained the sprayed surfaces nor did they cause an unpleasant odour or led to any complaints of adverse effects among the operators or sleepers at any stage of the study.

### Residual activity

**Figure 2:**
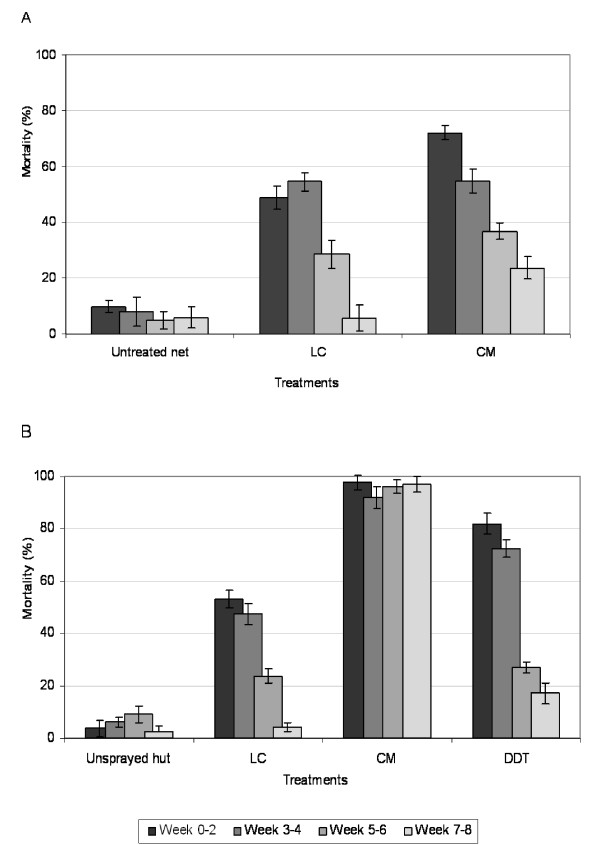
**Mortality of *Anopheles gambiae *freely entering the ITN and IRS treated huts on a fortnightly basis**. *Anopheles gambiae *mortality rates with 95% confidence interval, over fortnightly intervals during the hut trials with (A) insecticide treated nets (ITN) and (B) different indoor residual spray (IRS) treatments. Percentages are based on all mosquitoes collected from the rooms and veranda traps of huts. LC = Lambdacyhalothrin; CM = Chlorpyrifos methyl; DDT = Dichlorodiphenyltrichloroethane

Figure 2 shows the mortality of *An. gambiae *freely entering the ITN and IRS treated huts divided into fortnightly intervals during the 42 days of the trial. The proportion of *An gambiae *killed in huts with chlorpyrifos methyl ITN during weeks 0-2 was 73% after which mortality showed a progressive decline over the remaining six weeks (P < 0.001) (Figure 2A). By contrast, chlorpyrifos methyl IRS consistently killed more than 95% of pyrethroid resistant *An. gambiae *throughout the trial period. Mortality rates of *An. gambiae *in huts with DDT IRS and lambdacyhalothrin ITN or IRS decreased steadily between weeks 2-8 (P < 0.0001), presumably due to loss of activity and survival of *An. gambiae *carriers of *kdr *or other forms of pyrethroid resistance.

**Figure 3:**
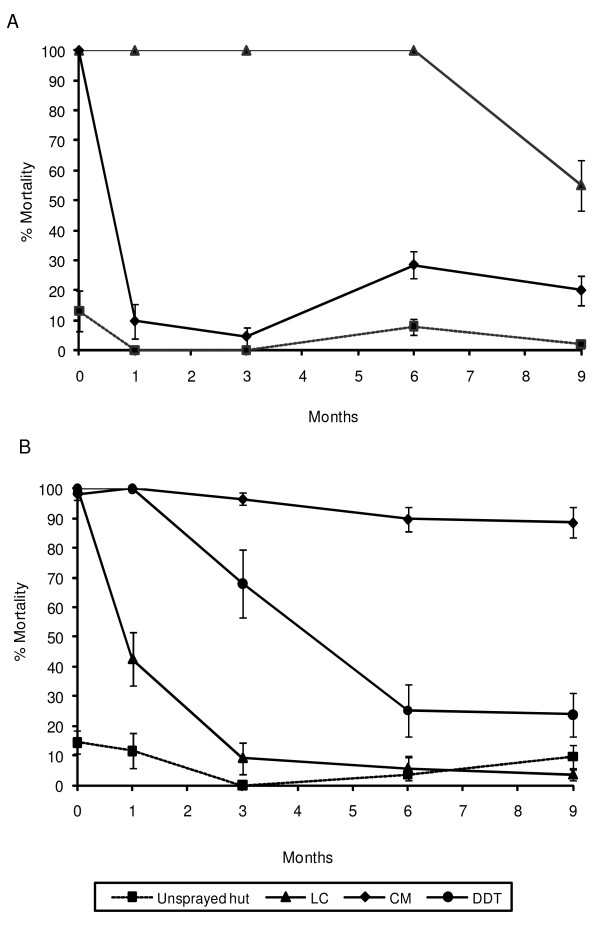
**Bioassay tests monitoring the residual activity of treatments using the susceptible *Anopheles gambiae *Kisumu strain**. Monitoring of the residual efficacy under WHO cone tests of (A) insecticide treated nets (ITN) and (B) different indoor residual spray (IRS) treatments against susceptible *An. gambiae *Kisumu strain in experimental huts at Ladji. LC = Lambdacyhalothrin; CM = Chlorpyrifos methyl; DDT = Dichlorodiphenyltrichloroethane.

Figure 3 gives results of bioassay tests conducted with susceptible *An. gambiae *Kisumu strain on (A) ITNs and (B) IRS cement wall surfaces. The residual activity of chorpyrifos methyl on ITN confirmed the rapid decline in effectiveness, with the mortality rate decreasing from 100% to 9.7% within one just month (P < 0.0001) (Figure 3A). Lambdacyhalothrin ITN remained highly active (100% mortality) for up to 6 months before showing a decrease in mortality at month 9 (P = 0.008).

The bioassays on chlorpyrifos methyl treated cement walls showed no loss of activity during the nine months of follow-up (P = 0.79) (Figure 3B). By contrast, decay of DDT and lambdacyhalothrin was evident on walls within the first month of spraying (P < 0.001).

## Discussion

Of the various groups of insecticide recommended by WHO for indoor residual spraying [23] the most cost-effective, DDT, is compromised by its negative environmental impact, and the most widely used, the pyrethroids, will surely accelerate the selection of resistance and undermine that other great tool for malaria prevention, the long-lasting pyrethroid-treated net [9,10,24]. The ideal compound for IRS would come from an entirely different class of insecticide to pyrethroids or organochlorines. The formulations of organophosphates and carbamates currently recommended for IRS are relatively short-lived [23] and this had limited their deployment by malaria control programmes. In the present study, the microencapsulated chlorpyrifos methyl applied as IRS treatment in Southern Benin killed almost all pyrethroid and DDT resistant *An. gambiae *that entered the huts and demonstrated a residual activity that lasted for at least nine months without showing any decay detectable by bioassay. This constitutes an insecticide formulation to rival DDT and pyrethroids in terms of residuality and cost effectiveness.

Chlorpyrifos methyl CS has potential for deployment in a variety of epidemiological settings. In areas of West Africa where pyrethroids or DDT no longer control *An. gambiae *because of resistance [9,10] chlorpyrifos methyl CS makes a promising alternative. Indeed, use of chlorpyrifos methyl CS or other long lasting cholinesterase inhibitor will be essential to keep malaria control or malaria elimination targets on track. With national malaria burdens continuing to fall [24] epidemics may become more frequent and IRS, as an emergency response may become more widely practised. Chlorpyrifos methyl CS is a less risky choice than DDT or pyrethroid if the local resistance status of *An gambiae *is unknown, because organophosphate resistance in this species is comparatively rare compared to pyrethroid resistance [17].

With its remarkable efficacy and residual activity it is essential to consider the issue of organophosphate resistance management from the outset. The most pragmatic approach to manage insecticide resistance is to rotate insecticides with differing modes of action between spray campaigns, although in reality sequential substitution of one unrelated compound for another once the former has failed is more the norm [25]. Ideally the use of pyrethroids should be constrained in order to preserve ITNs and LLINs. A promising partner insecticide to rotate with chlorpyrifos methyl in spray programmes is chlorfenapyr as this novel insecticide shows no cross resistance to OPs or pyrethroids in *An. gambiae *or *Cx. quinquefasciatus*, and has shown potential for IRS in areas where resistance to these two classes of insecticide has not yet been reported [26].

The short-lived residual activity of DDT in this trial (<2 months) compared to conventional wisdom (>6 months) might be due to the formulation obtained, to the cement substrate on which this insecticide was applied or to its residual activity perhaps being over stated [23]. Longer residual activity of DDT has been observed elsewhere on wooden walls in villages in Brasil [27] or Madagascar [28], but wood substrates are known to be rather benign to all types of insecticide. The study conducted in Southern Benin provides no reassurance that the activity shown by DDT would provide effective or sustainable control of malaria in areas where pyrethroid resistance involving *kdr *has become the norm.

There is a deterrent effect of lambdacyhalothrin-treated net against pyrethroid-resistant *An. gambiae *in Southern Benin and trustworthy higher personal protection than chlorpyrifos methyl because treatment could be rotated. For the lambdacyhalothrin IRS the result is less trustworthy because IRS treatments could not be rotated and the huts showed site effects with respect to mosquito numbers.

The hut trial also demonstrates the potential of chlorpyrifos methyl on nets for control of pyrethroid-resistant vectors. During the first few weeks, before chlorpyrifos methyl started to decay or wear off, the level of mortality was 100% against *An. gambiae*. With the advent of LLIN treatment technology there is scope for improving residual efficacy on nets using appropriate binding or incorporation technology.

## Conclusion

With its good safety profile, low mammalian toxicity and residual activity, chlorpyrifos methyl meets the profile of a cost-effective replacement for DDT or pyrethroids. The challenge of finding an environmentally acceptable alternative to DDT appears to be met. Adopting a IRS strategy that incorporates chlorpyrifos methyl will reduce the selective pressure generated by pyrethroids and help preserve the future of LLINs.

Despite the great promise shown by chlorpyrifos methyl it seems unlikely that the manufacturer, Dow AgroSciences, will deploy the long-lasting formulation in the near future, because of uncertainties in the vector control market. The comparatively small size of the market and the unpredictability of winning tenders is a major deterrent to companies not already engaged in malaria control. The manufacturer should be encouraged by international donors and technical authorities to pursue further development and evaluation against malaria indicators in endemic settings where *An. gambiae *is pyrethroid-resistant or IRS is being considered for malaria control.

## Competing interests

The authors declare that they have no competing interests.

## Authors' information

Raphael N'Guessan, Martin Akogbeto and Mark Rowland are all associated with the Pan African Malaria Vector Research Consortium, http://www.pamverc.org.

## Authors' contributions

RN co-designed and supervised the project, data analysis, drafted the manuscript.

PB, AO and JC conducted the field trials and bioassay testing.

MA Director of Centre de Recherche Entomologique de Cotonou, supervision and facilitation, reviewed and revised the manuscript.

MR coordination with WHO and manufacturers, co-designed the project, data interpretation, revised the manuscript.

All authors read and approved the final manuscript.
